# Health Resorts

**Published:** 1874-02

**Authors:** 


### HEALTH RESORTS.

### COLORADO.

(*From the Colorado Gazetteer.*)

Much has been said of the wonderful effects of a residence in Colorado, in the relief and cure of tubercular and pulmonary affections.

Probably one third of the present population of Colorado consists of reconstructed invalids. They have come here from all parts of the world in eager search of the fabled Fountain. Some came with gnawing and intractable dyspepsia; some with asthma or bronchitis; others had commenced "bleeding at the lungs," or were confirmed and hopeless victims of old fashioned "consumption." Many, it must be said, came too late. On the other hand, thousands, whose cases were considered hopeless, have here found permanent relief. This is especially true of asthmatics. For this class of patients the atmosphere of Colorado is almost a certain panacea.

Having given this subject especial investigation, and closely questioned the best medical authorities of the locality, we are led to conclude that, in cases of asthma, the cure is chiefly negative—an absence of the irritating and inciting causes, rather than any miraculous healing qualities inherent in atmosphere or climate. It is enough to know that, unless the convalescent returns again to his old haunts and habits, and to the original cause of his disease, this dreadfully distressing malady is here perfectly and permanently cured.

One of the most eminent medical authorities says:

"There are no fixed laws with respect to the best climate or situation for asthmatics; each case has its own law, which is only to be ascertained by experience. The principle to be acted upon is that there is a locality in which each martyr to this complaint will suffer less, and, perhaps, be entirely free from it; and the plan should be to make repeated triasl until the desired spot is found."

The disease, in its most aggravated and long seated forms, is relieved in nearly every instance, by a residence in the dry, salubrious atmosphere and perpetual sunshine of Colorado.

Consumptives, who come here before the ravages of the disease have been already too long unchecked, almost certainly recover; while others, who could exist elsewhere only in constant suffering and helplessness, are here enabled to pass the remainder of their days in comparative comfort, frequently regaining a considerable degree of vigor.

Dyspeptics also recover their lost powers of assimilation, and, by proper care, become robust.

Those afflicted with bronchitis and affections of the throat—many of whom have tried Minnesota, the West Indies, California and sea voyages in vain—become sound and well by a sojourn in Colorado.

The elements combining to produce such results are:

1. *Altitude.*—Colorado occupies the topographic centre, and summit of the continent. Its elevation ranges from four thousand feet in the Arkansas Valley, to ten thousand feet in the mountain parks, above the sea level.

The altitude most favorable for chronic invalids of the classes mentioned depends on the character of the disease, the age, temperament and habits of the patient, and the particular stage of advancement of the malady. In some cases of asthma the higher the elevation the more speedy and perfect the relief. A residence in the valley—as everything outside of the mountain proper is here termed—only ameliorates the severity of the paroxysms. On the other hand, cases of advanced pulmonary disease at the altitude of Georgetown (nearly nine thousand feet), would prove speedily fatal; while at Pueblo, on the Arkansas (four thousand five hundred feet), the patient would steadily improve, or, at least, live in comparative comfort.

Mere altitude is not the primary thing to be considered. The physiological effects of a residence in high regions are to hurry respiration, and accelerate the pulse and to compel an augmentation of the breathing capacity in order to provide the requisite quantity of oxygen from the rarefied air inspired. Hence, the danger to those who are far advanced with tubercular consumption, and to such as are subject to pulmonary hemorrhage. For the same reason, patients suffering from certain forms of heart disease are more injured than benefited by a removal to localities much more elevated than the one to which they have been accustomed.

For almost every other form of disease the increased activity imposed on the respiratory organs, by residence in high locations, is a direct and constant benefit. Nothing is better for a dyspeptic, or a sufferer from hepatic disorder, indigestion, or general torpor, than to make him breathe. Increase his respirations from sixteen to twenty-four per minute, and you give him a new existence. His blood circulates with equally increased rapidity, and is much more perfectly ærated; his appetite is increased; digestion and assimilation promptly responding to the increased demand and the increased action of the diaphragm, his biliousness oozes out through the pores of his skin, and he becomes a new being.

One of the curses of civilized life is the consummate stinginess with which people breathe. Here, whether in the "valley" or on the mountain heights—for even the bed of the Platte, at Denver, is a lineal mile higher above the sea level than New York or Philadelphia—one must breathe both more fully and more rapidly or die of suffocation

The result is a permanent increase of the breathing capacity, and the appetite keeps pace with the respiration.

2. *Climate.*—This varies considerably with the altitude, as well as the varying topography of the country. Almost any climate desired can be found within the limits of Colorado. In the southern portion, and in some of the sheltered valleys, there are days in midsummer when, for a few hours, the mercury ranges above 100° Fahr.; while, at the same moment there are heights in the mountains where one would be comfortable in the fur wrappings of a Laplander.

At Trinidad, near the southern borders, in the valleys of the Huerfano and Apishapa, and at Pueblo, on the Arkansas, the season opens considerably earlier than at Denver and other points north of the "Divide." Even at Colorado City, on the Fontaine qui Bouille, the springs are noticeably earlier than they are but a short distance further north and across the "Divide." The southern slope of the latter extending from its crest to the Arkansas—a distance of eighty miles—is so situated as to catch the perpendicular rays of the sun, and gather spring like warmth from them, while yet the snows lie unmelted on the northern slope.

Colorado City is situated at about the middle, and Pueblo at the base of the southern slope; while Denver is midway, and St. Vrain at the foot of the northern.

Located in latitude 37° to 41° north, between the extreme winters of the northern States and the enervating heats and humidity of the southern, Colorado enjoys an equable and desirable climate. Its winters are mild, comparatively little snow falling, except on the mountain ranges; and its summers are remarkably cool and bracing. There is about a month of each season during which, in the valley country, the mercury, at midday, ranges as high as at New Orleans; but one needs to keep on hand, in July, about the same clothing as is required at Christmas; and there are not half a dozen nights in the season when a pair of blankets to sleep under are, in any degree, uncomfortable. In the monntain towns gloves and overcoats are very convenient, even in dog days; and flannel underclothing should everywhere be worn the year round.

Chronic invalids are almost always benefited by a mere change of regimen, even if it be in some minor respects for the worse. If some change can be made from the humdrum of the eastern home to the fresh and novel life of a mountain country, with its more substantial bread, more virile, blood invigorating beef, its tempting mountain trout and juicy wild meat, the benefits are multiplied ten fold.

After what has been said, maladies for which this climate may be commended will readily suggest themselves:

Consumptives, in the first and second stages, may come to Colorado with assurance that whatever climate, natural hygienic surroundings, pure air and water, good food, grand scenery, romantic adventure and perpetual sunshine can do for an invalid here awaits them.

In the third and last stage, no combination of favorable influences and healthful climate, even with the aid of consummate medical skill, can avail further than to smooth the hopeless pathway to the inevitable end. Patients of this class can only be advised to come or stay according to the particular circumstances or preferences of each individual case.

That eminent English physician, Dr. Chambers, in his incomparable lectures on the *Renewal of Life*, gives this very sensible rule respecting the choice of climate:

"In choosing a home for your consumptive, do not mind the average height of the thermometer or its variations; do not trouble yourself about the mean rain fall; do not be scientific at all, but find out, from somebody's journal, how many days are fine enough to go out, forenoon and afternoon. That is the test you require, and by that you may be confidently guided."

Judged by this standard, Colorado is one of the most favored spots for a consumptive's refuge. There are not a score of days in any year in which invalids may not sit out of doors, ride or walk, with comfort. The nights are always cool, insuring refreshing sleep.

Another very important condition is the uniform dryness of the atmosphere. There is no "damp night air." One may sleep with doors and windows wide open, summer and winter, without once "taking cold." Even invalids sleep on the open plains, wrapped in a pair of blankets, but otherwise unprotected, with the most perfect impunity. Everything invites to out door life, and herein lies half the mystery of the "cures" which are credited to the country.

Of the results in dyspepsia, and all forms of indigestion, enough has already been said. Whatever will aid the consumptive will aid the dyspeptic; for the consumptive is first a dyspeptic, and, in fatal cases, always *starves to death.*

In patients afflicted with bronchitis the results are very flattering. Scarcely a case but is rapidly relieved.

With regard to that scourge of the eastern and northern States, catarrh, there is considerable difference of opinion. In a sweeping sense, whatever benefits the general health relieves this malady, and, in this regard, the country may be considered favorable for sufferers from catarrh. On the other hand the uniform dryness of the atmosphere is thought to aggravate many cases by favoring the formation of incrustations or concretions upon the inflamed mucous surface, and thus further irritating them. In a country where the rain fall is so scanty and the air and ground so dry, there is also necessarily experienced more irritation from dust; but this matter is much less annoying than it would be natural to expect. Some catarrh patients report immediate and thorough relief. Others assert that their cases are rather aggravated than improved. Doubtless very much depends upon the varying constitutional conditions and general habits of the different observers.

For all of scrofulous habit—to spinal complaint—there is no better climate.

And yet it is not enough that an invalid should come here to sit helplessly down, and wait for the climate alone to perform miracles in behalf of his restoration. All the inestimable aids of air, and food, and sunshine, and scenery, will be lost to such as allow themselves to mope in doors, and pine for home and former associations, and, perhaps, for the loss of enervating indulgences. Let them rather take to the saddle, explore the parks, shoot antelope on the plains, elk in the mountains, or feast on brook trout and salmon of their own catching. If they must have business, let them herd their own cattle, and live half the time in the saddle and the other half under a tent or on the naked sod.

There is another great army of sufferers, impossible to classify, who will find this country peculiarly adapted to their rapid and thorough restoration. We refer to those who, by close application, sedentary avocations, in door confinement, or nervous wear and tear from afflictions, financial reverses or social discordances, have become shattered in constitution, unfit for any kind of business, and tired of life. For such, here are new scenes, fresh experiences, intimate communion with nature in her most persuasive moods, rest from the world, and that best of all balms for hurt consciences and constitutions—sleep.

